# Supplementary material for: WordCommentsAnalyzer: A windows software tool for qualitative research
Source: F1000Res. 2018 Sep 4;7:536. Originally published 2018 May 3. [Version 2] doi: 10.12688/f1000research.14819.2 (PMC6137411; doi:10.12688/f1000research.14819.2)
Supplement: Supplementary file 3 [file f1000research-7-17614-s0002.tgz › b2a79496-4ada-47c4-9682-a99ce867836d.zip!/Eight Iranian Health Journals/Journal of Research in Rehabilitation Sciences _4.docx]

© Journal of Research in Rehabilitation Sciences . This work is licensed under CC BY-NC 4.0 (https://creativecommons.org/licenses/by-nc/4.0/).

Volume & Issue: دوره 13، شماره 1: 1396

Title: تعیین تأثیر اسپیلنت هالوفیکس بر زاویه هالوکس والگوس در افراد دارای دفورمیتی خفیف و متوسط هالوکس-والگوس در مقایسه با اسپیلنت شبانه: کارآزمایی بالینی دوسو کور

http://jrrs.mui.ac.ir/index.php/jrrs/article/view/2817

مقدمه: دفورمیتی (بدشکلی) هالوکس والگوس، از جمله رایج‌ترین اختلالات پا به شمار می‌رود که در آن، اولین مفصل متاتارسوفالنژیال به دلیل انحراف خارجی انگشت شست و انحراف داخلی اولین متاتارس، به ‌طور پیش‌رونده دچار نیمه دررفتگی می‌شود. متداول‌ترین مداخلات در موارد خفیف تا متوسط این دفورمیتی، استفاده از ارتزها می‌باشد که شامل دو نوع ارتز دینامیک جهت استفاده روزانه و ارتز استاتیک جهت استفاده شبانه است. هدف از انجام مطالعه حاضر، مقایسه تأثیر اسپیلنت دینامیک هالوفیکس و اسپیلنت شبانه بر زاویه هالوکس والگوس در افراد مبتلا به دفورمیتی خفیف و متوسط هالوکس والگوس بود. مواد و روش‌ها: در این پژوهش شبه تجربی، 34 بیمار که با شکایت هالوکس والگوس به متخصصان ارتوپدی اصفهان و شهرهای اطراف مراجعه کرده بودند، انتخاب شدند. این افراد در دو گروه 17 نفره که از نظر سن، جنسیت و شدت دفورمیتی یکسان بودند، قرار گرفتند. سپس به‌ طور تصادفی به یکی از گروه‌ها اسپیلنت شبانه و به گروه دیگر اسپیلنت هالوفیکس داده شد. زاویه هالوکس والگوس از طریق عکس رادیولوژی قدامی- خلفی پا در حالت تحمل وزن در ابتدای مطالعه و شش هفته پس از استفاده از ارتزها اندازه‌گیری گردید. یافته‌ها: استفاده از هر دو ارتز باعث کاهش زاویه هالوکس والگوس می‌شود، اما این کاهش از نظر آماری معنی‌دار نبود (05/0 < P). همچنین، تأثیر هر دو اسپیلنت بر کاهش زاویه هالوکس والگوس یکسان می‌باشد (97/0 = P). نتیجه‎گیری: استفاده شش هفته‌ای از اسپیلنت‌های شبانه و هالوفیکس، هیچ‌ کدام باعث بهبود زاویه هالوکس والگوس نمی‌شود.

Title: آیا الگو‌های حرکتی پایه در فوتبالیست‌های نوجوان پسر تحت تأثیر تمرینات عملکردی قرار می‌گیرند؟

http://jrrs.mui.ac.ir/index.php/jrrs/article/view/2838

مقدمه: الگو‌های حرکتی مناسب، برای پیشگیری از آسیب و اجرای حرکات ورزشی مهم می‌باشند. از این‌رو، استفاده از غربالگری حرکت عملکردی (Functional movement screen یا FMS) که نقش مهمی را در پیش‌بینی آسیب‌های ورزشی ایفا می‌کند، توسط بسیاری از متخصصان ورزشی در طراحی برنامه‌های تمرینی توصیه شده است. از طرف دیگر، سؤالاتی در مورد کاربرد این الگوها به عنوان وسیله‌ای برای ارزیابی اثربخشی تمرینی وجود دارد. هدف از انجام تحقیق حاضر بررسی این سؤال بود که آیا یک برنامه تمرینی عملکردی می‌تواند الگو‌های حرکتی پایه را تحت تأثیر قرار دهد؟ مواد و روش‌ها: 27 فوتبالیست‌ نوجوان پسر با دامنه سنی 14 تا 16 سال، بدون درد و سابقه آسیب در لایه‌های مختلف، به صورت تصادفی در دو گروه تجربی و شاهد تخصیص یافتند. گروه تجربی 10 هفته تمرینات عملکردی را اجرا نمودند. در مجموع، زمان اختصاص یافته به تمرین در هر دو گروه یکسان بود. قبل و بعد از اجرای تمرینات عملکردی، FMS در نمونه‌های دو گروه ارزیابی گردید. جهت مقایسه میانگین داده‌ها، آزمون‌های Independent t و تحلیل کواریانس در سطح معني‌داري 05/0 > P مورد استفاده قرار گرفت. یافته‌ها: اختلاف معنی‌داری در امتیاز FMS بین گروه‌های تجربی (49/1 ± 07/18) و شاهد (11/1 ± 07/16) مشاهده شد (001/0 > P و اندازه اثر = 71/0). نتیجه‌گیری: الگو‌های حرکتی پایه بررسی شده توسط FMS به دنبال یک مداخله تمرینی عملکردی، در فوتبالیست‌های نوجوان پسر بهبود یافت. بنابراین، شاید بتوان به مربیان و متخصصان ورزشی توصیه کرد که به جایگاه تمرینات عملکردی توجه ویژه‌ای نمایند. ضروری به نظر می‌رسد که در تحقیقات آینده، اثر تمرینات عملکردی بر سایر عوامل خطر آسیب‌ها نیز بررسی گردد.

Title: بررسی اثربخشی درمان کامپیوتری بر مهارت نامیدن بیمار مبتلا به زبان‌پریشی ناروان: یک مطالعه مورد- منفرد

http://jrrs.mui.ac.ir/index.php/jrrs/article/view/2839

مقدمه: آنومیا یا اختلال نامیدن، علامت اصلی زبان‌پریشی محسوب می‌شود. اگرچه درمان‌های مختلفی در زمینه نقص نامیدن وجود دارد، اما با توجه به ظهور تکنولوژی، یکی از شیوه‌های گسترش یافته امروزی، استفاده از کامپیوترها در درمان می‌باشد. هرچند اثربخشی درمان‌های کامپیوتری در زبان‌های مختلف بررسی شده است، اما به علت عدم بررسی این درمان‌ها در زبان فارسی، مطالعه حاضر با هدف تعیین اثربخشی درمان کامپیوتری توانا بر مهارت نامیدن بیمار مبتلا به زبان‌پریشی ناروان صورت گرفت. مواد و روش‌ها: این پژوهش به صورت طرح مورد- منفرد بود که در آن یک بیمار مبتلا به زبان‌پریشی ناروان با نقص نامیدن، جهت دریافت درمان کامپیوتری انتخاب گردید. همچنین، جهت بررسی اثر تعمیم، مجموعه‌ای از کلمات غیر آموزشی در نظر گرفته شد. داده‌ها با استفاده از روش تحلیل ديداري و اندازه اثر درصد داده‌های غیر همپوشان (Percentage of non-overlapping data یا PND) مورد تجزیه و تحلیل قرار گرفت. یافته‌ها: درمان کامپیوتری منجر به بهبودی توانایی نامیدن در مواجهه در بیمار گردید (100 = PND). همچنین، بهبودی در حفظ و تعمیم آیتم‌های غیر آموزشی در نتیجه این درمان حاصل شد (100 = PND). نتیجه‎گیری: نتایج مطالعه حاضر از تأثیر درمان کامپیوتری توانا بر بهبود مهارت نامیدن بیمار مبتلا به زبان‌پریشی ناروان به عنوان یک درمان مؤثر، فشرده، کم‌هزینه و مورد پذیرش فرد در زبان فارسی حمایت می‌کند.

Title: حساسیت و ویژگی فرم کوتاه آزمون تبحرحرکتی Bruininks–Oseretsky ویرایش دوم در کودکان دارای اختلال هماهنگی رشدی

http://jrrs.mui.ac.ir/index.php/jrrs/article/view/2847

مقدمه: ارزیابی مهارت‎های حرکتی و استفاده از یک ابزار مناسب برای تشخیص و شناسایی کودکان دارای اختلال هماهنگی رشدی (Developmental coordination disorder یا DCD)، در سنین پیش‎دبستانی حیاتی به نظر می‎رسد. بنابراین، هدف از انجام این پژوهش، ارزیابی حساسیت و ویژگی فرم کوتاه آزمون تبحر حرکتی Bruininks–Oseretsky ویرایش دوم، در کودکان پیش‎دبستانی بود. مواد و روش‌ها: تعداد 306 کودک پیش‎دبستانی (7-4 سال) در این مطالعه شرکت کردند. پس از آن، 30 کودک به عنوان اختلال هماهنگی رشدی انتخاب شدند. برای توانایی تشخیص اختلال هماهنگی رشدی آزمون، از تحلیل حساسیت و ویژگی آزمون با روش منحنی Receiver operating characteristic (ROC) استفاده شد. یافته‌ها: سطح زیر منحنی ROC برای حساسیت 91/0 و برای ویژگی 93/0 و به طور کلی سطح زیر منحنی 97/0 به دست آمد. نتیجه‎گیری: با توجه به نتایج این پژوهش، به نظر می‎رسد فرم کوتاه آزمون تبحر حرکتی Bruininks–Oseretsky ویرایش دوم، از حساسیت و ویژگی بالایی در کودکان پیش‎دبستانی برخوردار است و می‎توان از آن برای ارزیابی مهارت‎های حرکتی و شناسایی کودکان دارای اختلال هماهنگی رشدی استفاده کرد.

Title: بررسی تأثیر غوطه‌وری در آب سرد بر شاخص‌های عصبی- عضلانی خستگی ناشی از فعالیت سرعتی تکراری؛ بخش اول

http://jrrs.mui.ac.ir/index.php/jrrs/article/view/2813

مقدمه: با توجه به انجام انواع فعالیت‌های ورزشی طی مسابقات ورزشی، میزان خستگی و چگونگی کاهش آن متفاوت است. بر همین اساس، هدف از انجام مطالعه حاضر، بررسی میزان تغییرات مجذور میانگین ریشه (Root mean square یا RMS) و فرکانس متوسط (Median frequency یا MDF) عضلات چهارسر رانی و همسترینگ، به عنوان عوامل خستگی عصبی- عضلانی پس از غوطه‌وری در آب سرد به دنبال Repeated-sprint ability (RSA) بود. مواد و روش‌ها: به این منظور، 20 ورزشکار تمرین کرده، برای شرکت در این پژوهش انتخاب شدند. پس از انجام فعالیت سرعتی تکراری، 10 نفر از آزمودنی‌ها داخل آب سرد با دمای 14 درجه‌ سانتی‌گراد و 10 نفر دیگر در دمای اتاق به شکل غیر فعال قرار گرفتند. اندازه‌گیری الکترومایوگرافی (Electromyography یا EMG) حین حداکثر انقباض ارادی قبل و پس از انجام هر ست RSA و همچنین، پس از Cold water immersion (CWI) یا استراحت غیر فعال و پس از 24 ساعت انجام شد. یافته‌ها: پس از RSA در روز اول، کاهش معنی‌داری در RMS عضلات پهن جانبی [037/0 = PCWI، 010/0 = PCON (PControl)] و راست رانی (001/0 = PCON, CWI) رخ داد و همچنین، MDF عضله پهن جانبی تغییرات معنی‌داری (100/0 = PCWI و 850/0 = PCON) طی فعالیت سرعتی تکراری نداشت، در حالی که مقادیر MDF عضله راست رانی، به طور مستقیم پس از آب سرد (001/0 > P) و در آخرین ست روز دوم (030/0 = P) کاهش معنی‌داری را نشان داد. نتیجه‎گیری: این پژوهش نشان داد که غوطه‌وری در آب سرد، در مقایسه با ریکاوری غیر فعال، تأثیر معنی‌داری بر عملکرد عصبی- عضلانی ندارد.

Title: زمان‌بندی و شدت فعالیت عضلات شانه در حرکت پرتاب توپ هندبال در افراد بیمار دارای سندرم شانه و افراد سالم

http://jrrs.mui.ac.ir/index.php/jrrs/article/view/2842

مقدمه: آسیب شانه در ورزشکاران هندبال و سایر ورزش‌هایی که در آن‌ها پرتاب بالای سر وجود دارد، بسیار شایع است. عملکرد ضعیف یا نامناسب عضلات شانه، با بروز آسیب مرتبط است. هدف از انجام مطالعه حاضر، تعیین زمان‌بندی و شدت فعالیت عضلات شانه در پرتاب توپ هندبال در افراد مبتلا به سندرم درد شانه و افراد سالم بود. مواد و روش‌ها: زمان شروع فعالیت و شدت فعالیت عضلات ذوزنقه فوقانی، فوق خاری، دلتوئید میانی، خلفی، قدامی، سینه‌ای بزرگ، پشتی بزرگ و سه‌سر بازویی هنگام پرتاب پنالتی سریع توپ هندبال از بالای سر اندازه‌گیری شدند. همچنین، مقایسه بین گروهی با استفاده از آزمون Multivariate ANOVA در سطح معنی‌داری 050/0 > P انجام شد. یافته‌ها: در گروه سالم، شدت فعالیت عضلات ذوزنقه فوقانی و پشتی بزرگ، به ترتیب حدود 74/50 و 42/43 درصد بیشتر از آن در گروه بیمار بود، اما شدت فعالیت عضله دلتوئید میانی در افراد سالم، 05/38 درصد کمتر از بیماران بود. در افراد سالم، عضله فوق خاری، 5/23 میلی‌ثانیه زودتر از زمان شروع به حرکت پرتاب و عضله سه‌سر حدود 5/18 میلی‌ثانیه دیرتر از زمان شروع حرکت پرتاب، فعال شدند. در گروه بیماران نیز عضله فوق خاری زودترین (حدود 11 میلی‌ثانیه) و عضله سه‌سر با تأخیر (حدود 16/22 میلی‌ثانیه) فعالیت خود را آغاز نمودند. نتیجه‌گیری: هماهنگی عضلات شانه از نظر شدت فعالیت عضلانی و زمان شروع به فعالیت در افراد بیمار در حین پرتاب دچار اختلال می‌شود. از این رو، برنامه‌های تقویتی و کششی بدن‌سازی به منظور کاهش خطر ابتلا به سندرم درد شانه، به ورزشکاران توصیه می‌شود. همچنین، برنامه توان‌بخشی علاوه بر مهار درد، می‌تواند در اصلاح الگوی فعالیت عضلات ذوزنقه فوقانی، فوق خاری، پشتی بزرگ و دلتوئید در مراکز توان‌بخشی تمرکز یابد.

Title: تأثیر تحریک مستقیم جمجمه‌ای و تمرینات منتخب حرکتی بر مهارت‌های حرکتی ظریف در کودکان با اختلال طیف اتیسم

http://jrrs.mui.ac.ir/index.php/jrrs/article/view/2845

مقدمه: تحریک مستقیم جمجمه‌ای، یک تکنیک غیر تهاجمی است که یادگیری و اجرای حرکتی را تعدیل می‌کند. مطالعات پیشین نشان داده‌اند که تحریک مستقیم جمجمه‌ای بر روی قشر حرکتی اولیه، می‌تواند تقویت مهارت‌های حرکتی مختلف در آزمودنی‌های سالم را تسهیل کند؛ به ‌خصوص زمانی که به‌ صورت ترکیبی با تمرینات هدفمند انجام شود، اما تأثیر آن بر بهبود مهارت‌های حرکتی در اختلال اتیسم ناشناخته مانده است. هدف از انجام این پژوهش، بررسی تأثیر تحریک آندی و تمرینات منتخب حرکتی بر مهارت‌های حرکتی ظریف در کودکان با اختلال طیف اتیسم بود. مواد و روش‌ها: روش پژوهش، آزمایشی بود و از طرح پیش‌آزمون- پس‌آزمون با گروه شاهد استفاده شد. به این منظور، 18 کودک 14-6 ساله با اختلال طیف اتیسم با روش نمونه‌گیری در دسترس و با توجه به ملاک‌های ورود به پژوهش انتخاب و به ‌طور تصادفی در دو گروه مورد و شاهد گمارده شدند. 9 آزمودنی، تمرینات حرکتی را بعد از تحریک مغزی به‌ منظور بهبود مهارت‌های حرکتی ظریف انجام دادند. 9 آزمودنی دیگر، در جلسات تمرینات حرکتی به ‌طور مشابهی شرکت کردند؛ با این تفاوت که تحریک مغزی فقط برای 20 ثانیه اول اعمال می‌شد (گروه Sham). مهارت‌های حرکتی ظریف در قبل از مداخله و پس از 10 جلسه (بعد از مداخله) مورد ارزیابی قرار گرفت. یافته‌ها: تحریک آندی و تمرینات حرکتی، تأثیر معنی‌داری بر بهبود مهارت‌های حرکتی ظریف در کودکان مبتلا به اتیسم دارد (050/0 > P). نتیجه‌گیری: تحریک مغزی، می‌تواند مکمل سودمندی برای تمرینات حرکتی در کودکان اتیستیک باشد. اگر چه برای قطعیت یافته‌ها، به پژوهش‌هایی در گروه‌های بزرگ‌تر کودکان با سطوح مختلف علایم اتیسم و همچنین، قطب تحریکی متفاوت به ‌منظور ارزیابی استفاده عملکردی از تحریک غیر تهاجمی مغزی نیاز است.

Title: مروری نظام‌مند بر کارایی ربات‌های دستیار در عمل جراحی کاشت حلزون

http://jrrs.mui.ac.ir/index.php/jrrs/article/view/2789

مقدمه: جراحی کاشت حلزون، روشی تهاجمی برای ایجاد حس شنوایی است که خطراتی مانند آسیب حلزون گوش را به همراه دارد و لازم است تا حد امکان این خطرات کاهش یابد. کاشت حلزون‌ها در آینده سه هدف کلی شامل «کاهش آسیب به داخل حلزون گوش در حین عمل جراحی، بازدهی بیشتر عمل جراحی که به معنی کاهش بار تحریک الکتریکی برای تولید بلندی مناسب است و ورود عمیق‌تر به حفره اسکالا تیمپانی برای رسیدن به اعصاب حلزونی مربوط به فرکانس‌های پایین‌تر» را دنبال می‌کند. برای رسیدن به این اهداف، نیاز به دقت بالایی است که امروزه استفاده از ربات دستیار در جراحی، راه‌حل مناسبی به نظر می‌رسد. هدف از انجام مطالعه حاضر، بررسی کارایی سیستم‌های رباتیک در عمل جراحی کاشت حلزون بود. روش: در این پژوهش، مقالات پایگاه‌های علمی PubMed، ScienceDirect و Google Scholar در بازه زمانی سال‌های 1980 تا 2017 با استفاده از واژگان مرتبط با موضوع، مورد برررسی قرار گرفت و مقالات با توجه به معیارهای ورود و خروج انتخاب گردید. یافته‌ها: مطالعه بر روی سه سیستم رباتیک تمرکز نمود؛ اولین روش، هدایت مغناطیسی بود که در آن از کاشت حلزونی که نوک پروتز آن خاصیت مغناطیسی دارد، برای ورود به حلزون گوش استفاده می‌شود. در این روش یک آهنربای کنترلی چرخان در نزدیکی سر افراد جهت هدایت مغناطیسی پروتز قرار می‌گیرد. دومین سیستم بر روی تعیین تغییرات زاویه ورود آرایه الکترودی به داخل حلزون، به وسیله آرایه‌های الکترودی ورودی کنترل شده تمرکز دارد و سیستم دیگر، تهاجم جراحی را به وسیله از بین بردن نیاز به ماستوئیدکتومی و تعویض آن با روش تونل مستقیم (دسترسی مستقیم حلزونی)، کاهش می‌دهد. نتیجه‌گیری: سیستم‌های رباتیک موجب کاهش انرژی مورد نیاز برای ورود آرایه الکترود، ورود عمیق‌تر آرایه به حلزون گوش برای بهبود عملکرد کاشت حلزون و کاهش آسیب به داخل حلزون گوش در حین عمل جراحی می‌شود. به دنبال موارد مذکور، درک گفتار در حضور نویز و کیفیت صدا برای کاربران بهبود خواهد یافت.
